# Supplementary material for: Food Addiction among Female Patients Seeking Treatment for an Eating Disorder: Prevalence and Associated Factors
Source: Nutrients. 2020 Jun 26;12(6):1897. doi: 10.3390/nu12061897 (PMC7353200; doi:10.3390/nu12061897)
Supplement: Supplementary file 1 [file nutrients-12-01897-s001.pdf]

Table S1. Description of the sample according to ED (n=195).

	Entire sample (N=195)	AN-R *(n=65)*	AN-BP *(n=33)*	BN *(n=82)*	BED *(n=15)*
		n (%) or m (sd)			
**Sociodemographic characteristics**					
Age (years)	23.1 (7.4)	21,4 (5.9)	22.2 (6.7)	24.0 (8.3)	27.1 (8.1)
**Eating disorders characteristics**					
Recurrent episodes of binge eating (yes)	114 (58.5%)	0 (0.0%)	17 (51.5%)	82 (100%)	15 (100%)
Age of disease onset (years)	15.9 (5.0)	17,4 (5.1)	14.4 (3.7)	15.4 (3.7)	15.6 (10.1)
Disease duration (years)	7.2 (7.6)	4.0 (5.1)	7.8 (8.0)	8.6 (8.3)	11.5 (8.0)
Severity of ED (MROAS total score)	6.4 (2.0)	5.9 (1.8)	5.2 (1.8)	7.0 (1.9)	7.9 (1.5)
Dimensions associated with ED (EDI-2)					
Ineffectiveness	13.4 (7.0)	11.5 (6.1)	13.8 (7.5)	14.4 (7.2)	14.5 (7.1)
Interoceptive awareness	13.6 (6.8)	10.5 (6.0)	16.8 (5.9)	14.7 (6.9)	13.4 (6.4)
Asceticism	8.5 (4.5)	7.5 (4.1)	10.1 (4.1)	9.2 (4.8)	6.3 (3.4)
Drive for thinness	15.3 (5.1)	13.6 (5.8)	16.0 (5.2)	16.8 (4.0)	12.9 (4.3)
Bulimia	8.2 (6.7)	1.6 (2.6)	7.6 (6.1)	12.7 (4.7)	12.9 (4.7)
Body dissatisfaction	18.0 (7.2)	13.4 (6.8)	19.0 (6.2)	20.5 (6.2)	22.1 (6.2)
Perfectionism	7.3 (4.5)	6.7 (4.1)	8.6 (4.4)	7.2 (4.6)	7.0 (4.9)
Interpersonal distrust	7.7 (4.5)	7.0 (3.7)	9.4 (4.6)	7.6 (5.0)	7.2 (4.6)
Maturity fears	7.9 (5.9)	7.5 (6.0)	8.7 (5.8)	8.0 (6.0)	7.5 (5.3)
Impulse regulation	8.4 (6.6)	6.5 (5.3)	9.4 (6.8)	9.5 (7.1)	8.5 (7.0)
Social insecurity	9.9 (4.8)	8.9 (3.8)	10.7 (5.5)	10.5 (5.1)	9.3 (4.7)
**Comorbidities (current or past)**					
Mood disorders (MINI)	156 (80.0%)	46 (70.8%)	28 (84.8%)	69 (84.1%)	13 (86.7%)
Anxiety disorders (MINI)	141 (72.3%)	42 (64.6%)	28 (84.8%)	60 (73.2%)	11 (73.3%)
Psychotic syndrome (MINI)	12 (6.2%)	3 (4.6%)	2 (6.1%)	7 (8.5%)	0 (0.0%)
Addictive disorders (MINI and MIDI)	90 (46.2%)	24 (36.9%)	18 (54.5%)	41 (50.0%)	7 (46.7%)
ADHD in childhood (WURS-C)	66 (33.8%)	10 (15.4%)	13 (39.4%)	36 (43.9%)	7 (46.7%)
**Impulsivity**					
UPPS-Urgency	10.5 (3.0)	9.9 (2.8)	10.5 (3.2)	11.0 (2.9)	11.0 (3.2)
UPPS-Premeditation (lack)	7.5 (2.5)	6.8 (2.1)	6.8 (2.3)	7.9 (2.5)	9.4 (2.7)
UPPS-Perseverance (lack)	7.4 (2.9)	6.6 (2.2)	6.7 (2.4)	7.8 (2.8)	10.6 (4.3)
UPPS-Sensation seeking	9.8 (3.2)	9.2 (3.0)	10.7 (3.7)	9.9 (3.0)	10.3 (2.8)
**Temperament Comorbidities (current or past)**					
TCI-Novelty seeking	40.8 (19.3)	33.4 (16.1)	40.0 (16.9)	43.9 (20.2)	58.0 (18.0)
TCI-Harm avoidance	74.2 (20.6)	75.9 (20.2)	69.5 (21.5)	73.8 (21.0)	78.7 (17.4)
TCI-Reward dependence	60.4 (17.5)	60.7 (16.2)	54.4 (17.3)	61.6 (18.4)	65.5 (17.4)
TCI-Persistence	72.6 (28.7)	79.4 (26.0)	89.1 (17.4)	63.4 (30.4)	57.3 (26.0)
**Attachment**					
RSQ-Secure	2.7 (0.6)	2.7 (0.5)	2.7 (0.6)	2.7 (0.6)	2.5 (0.6)

RSQ-Fearful	2.9 (0.6)	2.7 (0.6)	3.0 (0.6)	2.9 (0.6)	3.1 (0.5)
RSQ-Preoccupied	2.6 (0.7)	2.5 (0.6)	2.3 (0.7)	2.7 (0.6)	2.7 (0.6)
RSQ-Dismissing	3.3 (0.8)	3.1 (0.8)	3.6 (0.7)	3.2 (0.8)	3.4 (0.8)
**Life events**					
History of physical abuse	20 (10.3%)	6 (9.2%)	5 (15.1%)	6 (7.3%)	3 (20.0%)
History of sexual abuse	27 (13.8%)	6 (9.2%)	7 (21.2%)	11 (13.4%)	3 (20.0%)

%: percentage; m: mean; sd: standard deviation; ADHD: attention-deficit/hyperactivity disorder; AN-BP: anorexia nervosa binge-eating/purging type; AN-R: anorexia nervosa restricting type; BED: binge eating disorder; BN: bulimia nervosa; ED: eating disorder; EDI: Eating Disorders Inventory; MIDI: Minnesota Impulsive Disorders Interview; MINI: Mini International Neuropsychiatric Interview; MROAS: Morgan–Russell Outcome Assessment Schedule; RSQ: Relationship Scales Questionnaire; TCI: Temperament and Character Inventory; UPPS: Impulsive behavior scale; WURS-C: Wender Utah Rating Scale-Child; YFAS: Yale Food Addiction Scale.
